# An Extensive Review of Natural Polymers Used as Coatings for Postharvest Shelf-Life Extension: Trends and Challenges

**DOI:** 10.3390/polym13193271

**Published:** 2021-09-25

**Authors:** Maricarmen Iñiguez-Moreno, Juan Arturo Ragazzo-Sánchez, Montserrat Calderón-Santoyo

**Affiliations:** Laboratorio Integral de Investigación en Alimentos, Tecnológico Nacional de México, Instituto Tecnológico de Tepic, Av. Tecnológico 2595, Tepic 63175, Nayarit, Mexico; mari.moreno2312@gmail.com

**Keywords:** polysaccharides, proteins, lipids, composite coatings, postharvest protection, edible films

## Abstract

Global demand for minimally processed fruits and vegetables is increasing due to the tendency to acquire a healthy lifestyle. Losses of these foods during the chain supply reach as much as 30%; reducing them represents a challenge for the industry and scientific sectors. The use of edible packaging based on biopolymers is an alternative to mitigate the negative impact of conventional films and coatings on environmental and human health. Moreover, it has been demonstrated that natural coatings added with functional compounds reduce the post-harvest losses of fruits and vegetables without altering their sensorial and nutritive properties. Furthermore, the enhancement of their mechanical, structural, and barrier properties can be achieved through mixing two or more biopolymers to form composite coatings and adding plasticizers and/or cross-linking agents. This review shows the latest updates, tendencies, and challenges in the food industry to develop eco-friendly food packaging from diverse natural sources, added with bioactive compounds, and their effect on perishable foods. Moreover, the methods used in the food industry and the new techniques used to coat foods such as electrospinning and electrospraying are also discussed. Finally, the tendency and challenges in the development of edible films and coatings for fresh foods are reviewed.

## 1. Introduction

Global demand for fresh and minimally processed fruits and vegetables is increasing in parallel with population growth [1]. These foods are essential in the human diet due to the nutritional benefits associated with their consumption. However, these foods have a relatively short postharvest life, ranging from a few hours to a few weeks at ambient temperature. They remain as living tissues because their physiological and biochemical process continues until the consumption time [1,2]. The weight loss in fruits and vegetables during the postharvest stage by transpiration is one of the most important troubles to maintain their quality. This physiological parameter results in textural changes such as bruising development. Fruit softening during the storage is also attributed to the deterioration of the cell wall components, caused mainly by the enzymatic activity [3]. The losses of fruits and vegetables caused by microorganisms along food chain production can reach more than 25% of the total production in industrialized countries, and over 50% in developing countries if postharvest handling and storage conditions are not optimal [3,4]. Hence, the appropriate postharvest handling, packaging, transportation, and storage practices are highly important to reduce postharvest losses of these foods [1,5]. Among them, packaging is the most important step for the transportation of foods from the industry to the retail store [6].

Postharvest treatments with conventional synthetic waxes such as polyethylene and petroleum waxes or with chemical fungicides have been used for many years to control postharvest decay and extend the fruit shelf-life. However, the continuous application of these treatments has negative effects on the environment and human health. The increasing environmental awareness, the restrictions on the use of agrochemicals, and the growing consumer demand for healthy fresh foods have intensified the exploration of new preservation technologies based on abundant, low-cost, renewable, and biodegradable alternatives [3,7]. In agreement with this, the use of edible coatings (ECs) has emerged as an effective and eco-friendly alternative to extend the shelf-life of fresh foods in recent years [8,9]. An EC is a thin layer of an edible material with filmogenic properties applied directly on a food surface in its liquid form [8]. One of the main interests in coating design is the inclusion of substances with antimicrobial activity within polymeric matrices. Non-toxic antifungal compounds incorporated in EC can control fungal decay, which is one of the main causes of postharvest losses of fruit and vegetables [3].

## 2. Characteristics of Edible Coatings to Extend the Shelf-Life of Fruit and Vegetables

The main characteristic of food-grade coatings is that can be eaten as part of the whole product, being particularly important in ready-to-eat fruits and vegetables. Therefore, the composition of ECs must comply with the regulation of the food product in each country [10]. The most important properties of edible films and coatings are the barrier properties to water vapor, gases, and compound migration, their capability for physical and mechanical protection, and improved food appearance [2]. Additionally, coatings can act as carriers of biocontrol agents and a wide variety of bioactive and/or functional compounds, such as antimicrobials, antioxidants, anti-browning agents, volatile precursors, nutrients, flavoring, and coloring compounds [11,12,13]. The addition of these compounds promotes the coatings’ functional performance, enhancing the stability, quality (reduces biochemical deterioration, enzymatic browning, and development of off-flavors), and safety of the foods [8,9].

The effectiveness of an EC to protect fruits and vegetables greatly depends on the biopolymers’ wettability and uniformly of the coated food surface. This behavior is influenced by the fruit/vegetable surface properties and by the chemical composition of the coating-forming solution: the polymeric substance, the presence of different compounds such as plasticizers, surfactants, crosslinker agents, functional compounds, and others [14]. Moreover, the EC effectiveness is closely related to tensile properties. Mechanical resistance is important to prevent the coating fracture and to protect the food product from mechanical factors and the physical damage caused by impact, pressure, or vibrations during storage [3]. The addition of plasticizers and emulsifiers (or surfactants) to the coating solutions improves the flexibility, extensibility, and/or stability of the structure of the coating [2]. 

Otherwise, ECs should regulate the mass transfer in the food to prevent moisture loss in the products (weight loss) and any change in texture, flavor, and/or appearance [15]. Coatings must provide an adequate gas barrier (low oxygen permeability values) because the respiration process increases ethylene production, accelerating the consumption of sugars and other compounds, and causing senescence [16]. However, to prevent anaerobic respiration and fermentative process, moderate barriers with a certain degree of oxygen and carbon dioxide permeability are needed for the respiration of living tissues [17]. In this sense, depending on the different respiration rates of the food, a different minimum oxygen transfer rate may be needed to avoid undesirable metabolic changes [3]. The good adherence and extensibility of the coatings are important factors to considerer because a coating should improve the appearance and attractiveness of the coated fruit or vegetable. 

**Figure 1:**
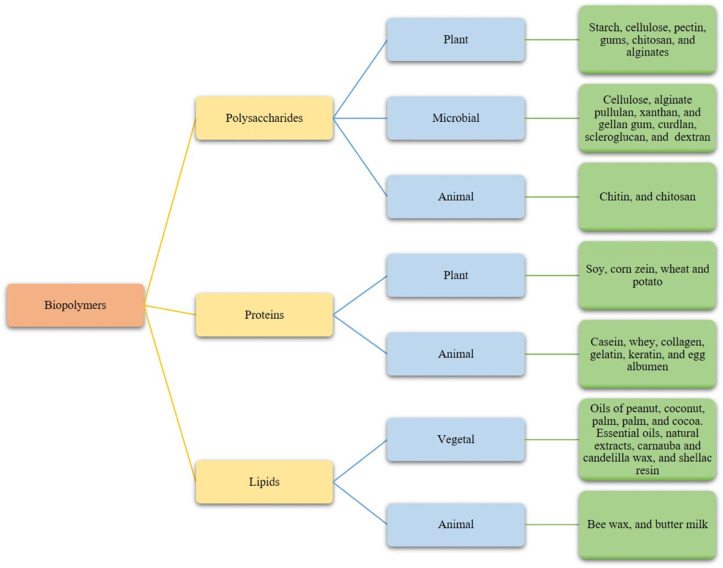
Biopolymers used as natural edible films and coatings and their sources.

On the other hand, is important to consider that some active ingredients might change the organoleptic profile of the coated product, causing undesirable odors or modifications in the functional properties. Some active compounds, such as essential oils (EOs), may cause toxicity in plant cells at high concentrations, or lose their functionality when reacting with food components or external factors [18]. The characteristics, efficiency, and stability of ECs depend on various chemical and physical factors, such as the chemical structure of the polymeric matrix, pH, viscosity, thickness, cross-linking degree, and processing conditions, as well [19]. Lipids, proteins, and polysaccharides of renewable sources are widely used alone or in combination in the coatings forming solutions (Figure 1) [20]. ECs based on proteins and carbohydrates usually have better cohesive, flexible, and gas-barrier properties than wax coatings [21]. 

## 3. Composition of Edible Coatings

### 3.1. Polysaccharide-Based Coatings

Polysaccharides are the most used components to coat fruits because of their microbial and physical stability over time, low cost, and great film-forming properties. Polysaccharides are compatible with a broad range of functional compounds and additives to improve their filmogenic properties [3] (Table 1). The main biopolymers used to develop ECs are starch, cellulose, pectin, gums, chitosan, and alginates [9,20], pullulan, xanthan, and gellan gum [22] (Figure 1). 

#### 3.1.1. Cellulose and Cellulose Derivates-Based Films and Coatings

Cellulose is a homopolymer synthesized by the polymerization of glucose residues from a substrate as UDP-glucose linked by β-1,4-O-glycosidic bonds forming a β-1,4-D-glucan. Cellulose chains have a strong tendency to self-associate by H-bonds to form insoluble and crystalline fibrils. In nature, the degree of polymerization and the diameter of cellulose microfibrils depends on the source and age of the tissue. Cellulose is the most abundant and renewable polymer resource [38,39]. This biopolymer is the main constituent of higher plants being wood and cotton the principal sources for industrial processes because it constitutes 40–50% and nearly 90% in wood and cotton, respectively. Moreover, nano-, microcrystalline, and nanofibrillar cellulose can be obtained through a sustainable process from sugarcane [40], cassava [41], and agave bagasse [42], respectively. Cellulose is also produced by the acetic acid bacterium and is found in the cell walls of fungi and green algae [43]. Its purity varies according to the source of obtention; bacterial cellulose has higher purity, mechanical strength, crystallinity, and hydrophilicity [44]. Its high hydrophilicity makes cellulose insoluble in aqueous solutions and polar solvents. Therefore, the etherification of the hydrogen atom in the hydroxyl groups of cellulose is used to obtain derivates water-soluble [45]. 

The main cellulose derivates used for coatings are methylcellulose, hydroxypropyl methylcellulose, hydroxyethylcellulose, and carboxymethyl cellulose [23,46]. They have been mixed with other polysaccharides, such as starch, to improve their water solubility. [46]. Hydroxypropyl cellulose and methylcellulose films and coatings are efficient barriers to oxygen, carbon dioxide, and lipids, but with poor resistance to water vapor transport. Although, the water vapor barrier properties can be improved by adding hydrophobic materials such as lipids into the film- or coating-forming solution [47]. Cellulose derivates have been widely used to develop films and coatings [48] due to their great film-forming properties, high availability, biodegradability, and biocompatibility [23]. 

#### 3.1.2. Starch-Based Films and Coatings

Starch is a natural, renewable, and biodegradable polymer produced by many plants, representing the most significant form of carbon reserve [20,49]. It is the second most abundant material in nature, and it is found in plant roots, stalks, crop seeds, and staple crops such as rice, corn, wheat, tapioca, and potato [50,51]. Starch is composed of two D-glucose homopolymers, amylose, and amylopectin; moreover, starch granules can have protein and lipids residues [50,52]. Amylose is a linear molecule, in which glucosyl monomers are joined by α-bonds (1→4). Otherwise, amylopectin is the highly branched component of the starch and is responsible for starch film-forming properties [52]. It is formed through chains of α-D-glucopyranosyl residues linked mainly by α-bonds (1→4), but around 5–6% are joined by α-bonds (1→6), which induce the formation of branches [50,52]. The differences in the structure and molecular weight between amylose and amylopectin lead to differences in their molecular properties and film-forming properties [20].

The starch used as is extracted from the plants is called “native starch”; in this stage, it has limited industrial applications due to its insolubility in cold water, hygroscopicity, undesirable texture, retrogradation, etc. Therefore, the starch is chemical, enzymatical, and physically modified to reach specific properties and is called “modified starch” [51,53]. The application of films and edible coatings based on starch is extensive because they have no smell or taste, are transparent, and have an oil-free appearance [28,54]. However, due to their hydrophilicity, starch-based films and coatings have poor water vapor barriers. To improve this drawback, hydrophobic substances such as oleic acid and shellac are added to decrease the respiration rates of the coated fruit, contributing to the reduction of weight loss [55]. Otherwise, to improve the water solubility and wettability properties, hydrophilic plasticizers such as glycerol and sorbitol are added [56,57]. In addition, starch coatings have poor mechanical properties, which are enhanced by the addition of other polysaccharides such as chitosan [52]. Obtaining starch coatings is necessary to gelatinize the starch granules in an excess of water (>90%, *w*/*w*), to obtain a homogeneous film-forming solution. This process breaks the amylopectin matrix and releases the amylose by water diffusion through the granules, promoting the melting of the starch crystallites [58]. The starch packaging can be obtained by dry and wet processes. Starch extrusion is possible due to its thermoplastic properties. In the extrusion, the starch is heated above its glass transition temperature in the presence of low water content. Otherwise, in the wet process, the polymers are solubilized and then the film-forming solution is dried. Usually, the wet process is chosen to form edible preformed films, or to apply coatings by dipping, brushing, or spraying onto food products [59]. However, dry methods are more easily implemented at the industrial level [60]. 

#### 3.1.3. Chitin and Chitosan-Based Films and Coatings

Chitin is a natural and abundant mucopolysaccharide, the structural component of crustaceans, insects, and fungal mycelia, consists of 2-acetamido-2-deoxy-β-D-glucose linked by β-bonds (1→4). Chitosan is the *N*-deacetylated derivate of chitin obtained in the presence of concentrated alkali [61]. Chitin and chitosan are similar to cellulose in their high insolubility degree and low chemical reactivity. Chitosan solubility depends on *N*-acetylation degree and molecular weight; however, it can be dissolved in acid solutions (pH < 6.3), even at concentrations above 2% (*w*/*v*). Solubility, appearance, rheological properties, among other properties of chitosan properties also depend on the *N*-acetylation degree [62].

Chitin and chitosan are very important due to their high nitrogen content (6.89%), biocompatibility, biodegradability, non-toxicity, adsorption, and chelator properties. For these reasons, it has been widely used in the biomedical, pharmaceutical, and food industry [61,63]. Chitosan films and coatings possess good barrier properties to control gas exchange (O_2_ and CO_2_), this is very important in fresh foods due to its contribution to delay the ripening process. Moreover, chitosan has great antimicrobial properties against several fungi, yeasts, bacteria, and viruses [20,64,65,66]. 

A disadvantage of the chitosan coatings is their high permeability to water vapor limiting their use in food products [67]. For this reason, several strategies have been proposed to improve the functional properties of chitosan films and coatings. For example, modifications of the deacetylation degree, pH, different solvents, and the use of plasticizers, surfactants, proteins, polysaccharides, or lipids can improve its functional coating properties [68,69]. The addition of EOs improves the water vapor permeability and imparts additional antimicrobial and antioxidant properties [70]. Moreover, to prevent dissolving and/or swelling and improve the properties of chitosan-based coatings, the formulations have been added with several reagents such as genipin, glutaraldehyde, formaldehyde, cinnamaldehyde, sodium trimetaphosphate, and ferulic acid [70,71].

#### 3.1.4. Pectin-Based Films and Coatings 

Pectin is the most abundant polysaccharide in the cell wall and middle lamella of plants, representing ~30% of the dry basis [72,73]. However, only a few plants are used to obtain commercial pectin in the food industry, citrus and apple peels being the main materials used for its extraction [20,74]. Pectin is composed of at least three polysaccharide domains: homogalacturonan (HGA), rhamnogalacturonan-I, and rhamnogalacturonan-II. HGA is the major component of pectin, which is a linear chain of galacturonic acid linked by α-bonds (1→4) [73]. The carboxyl groups of the galacturonic acid units are esterified with methanol, and occasionally, partially acetyl-esterified. Depending on its esterification degree with methanol, pectin is classified as high methoxyl pectin (HM, >50% esterified carboxyl groups), or low methoxyl pectin (LM, <50% esterified carboxyl groups) [75]. 

Pectin can form insoluble hydrogels in presence of di- or trivalent cations. However, the degree and pattern of esterification along the polysaccharide chain determine the gelling properties. Other parameters that should be considered in the pectin gelling process are temperature, pH, and solutes. HM pectin form gels at pH < 3.5 in the presence of more than 55% (*w*/*v*) co-solutes, as sucrose, due to the formation of entanglements, hydrophobic interactions, and H-bonds. The formation of water-insoluble gels from LM pectin occurs in the presence of positive divalent ions over a wide range of pH values with or without co-solutes [76]. The interaction of carboxyl groups of LM pectin with Ca^2+^ develops a structure similar to the egg box described to alginate [77]. The gelling and mechanical properties, stability, and response to chemical and physical conditions of pectin depending on the source of the extraction and method applied to its obtention [78]. 

#### 3.1.5. Alginate-Based Films and Coatings 

Alginate is a polysaccharide rather abundant in nature, a structural component in marine brown algae (Phaeophyceae, mainly *Laminaria*) [57,79]. It is also produced by two genera of bacteria, *Pseudomonas* and *Azotobacter* [80]. Alginate is the salt of alginic acid, which is a linear copolymer of (1→4) β-D-mannuronic (M) and α-L-guluronic (G) acid. These acid residues are organized in sections of M or G residues, called M or G blocks, as well as sections of M and G residues, referred to as MG blocks. The proportion and distribution pattern of M:G residues vary according to the algae species [81].

Alginate is widely used in the food industry due to its facility to bind to divalent or trivalent cations and form gels [82]. The divalent ions establish an association between M and G blocks, resulting in a stable and ordered three-dimensional network known as the ‘‘eggbox’’ model [83]. The association with ions occurs by the electrostatic interaction between the negatively charged carboxylate group of alginate and the ions in the cross-linking solution [84]. The affinity to the cations increase in the order Mg^2+^ < Ca^2+^ < Sr^2+^ < Ba^2+^ [82]. The cross-linking process with polyvalent cations has been used to improve the barrier properties, mechanical resistance, cohesiveness, and stiffness of alginate films and coatings [20]. Moreover, the addition of vegetable oils such as rapeseed oil, coconut oil, and hazelnut oil, or apple puree improved the mechanical and thermal properties of sodium alginate films [85].

#### 3.1.6. Xanthan Gum-Based Films and Coatings

Xanthan, a natural gum, is an extracellular heteropolysaccharide produced by the bacterium *Xanthomonas campestris* [86,87]. Xanthan gum consists of repeated pentasaccharide units formed by two glucose, two mannose, and one glucuronic acid. Its main chain consists of β-D-glucose units linked at the 1 and 4 positions, being identical to that of cellulose. The variations in the fermentation process, produce variation in the molecular weight in ranges from 2 × 10^6^ to 20 × 10^6^ Da [87]. Xanthan gum coatings have high moisture content and water vapor permeability; the incorporation of gelatin or starch reduces these values and improves the mechanical and thermal properties and thermal stability of the films [88,89]. However, phase separation into xanthan gum-gelatin and xanthan gum-starch films has been reported [90,91]. To solve this drawback, the oxidation of xanthan gum in the C_2_-C_3_ bond results in the formation of aldehyde groups. These groups can crosslink with ε-amino groups of lysine or hydroxylysine side groups of protein by Schiff’s base formation, enhancing the mechanical properties of the films and coatings [88,92]. 

#### 3.1.7. Pullulan-Based Films and Coatings

Pullulan is an extracellular water-soluble homopolysaccharide of glucose obtained from the fermentation broth of the fungus *Aureobasidium pullulans*. Pullulan is an amorphous slime matter consisting of maltotriose repeating units joined by α-1,6 bonds. The internal glucose units within maltotriose are connected by an α-1,4-glycosidic bond. The molecular weight of pullulan has considerable variety, ranging from 4.5 × 10^4^ to 6 × 10^5^ Da, greatly affected by culture parameters [93,94,95]. High-molecular-weight pullulan films exhibit better performance on physical properties, particularly water resistance [96].

Pullulan easily dissolves in water to form a stable and viscous solution forming a solution stable over a wide range of pH and temperatures. This polysaccharide forms thin and biodegradable films, which are transparent and highly adhesive with low oxygen permeability, low toxicity, and good mechanical properties. Its high solubility is the main disadvantage of pullulan coatings; therefore, cross-linking agents such as glutaraldehyde and *κ*-Carrageenan have been used to enhance the tensile strength and reduce the hydrophilicity of pullulan films and coatings [96,97].

#### 3.1.8. Gellan Gum-Based Films and Coatings 

Gellan is an anionic extracellular bacterial polysaccharide produced by *Sphingomonas elodea*, also known as *Pseudomonas elodea* [98,99,100]. Gellan is a linear polymer with a tetrasaccharide repeating sequence, consisting of two residues of β-d-glucose, one of β-d-glucuronate, and one of α-l-rhamnose [99]. Low variations in its molecular weight have been obtained when factors such as the pH, temperature, aeration, agitation, and release from a fluid broth are strongly controlled [100]. Gellan films and coatings are transparent, biocompatible, biodegradables, non-toxic, with thermal and acid stability, and resistant to enzymatic activity [98]. Commercially the gellan gum is in two presentations: deacylated or low acyl gellan gum and acyl or acylated gellan gum [100]. The deacylated gellan gum forms a rigid and brittle hydrogel upon cooling at 40 °C, while the acyl gellan gum forms a soft and flexible hydrogel upon cooling at 65 °C [101,102], limiting its application in the development of food packaging [98]. Therefore, it was suggested that flexibility and hardness can be better controlled through blends with natural and synthetic polymers [103]. The negative charge of gellan allows the production of polyelectrolytes with other oppositely charged polymers such as chitosan and cationic guar gum [104,105]. The gelling properties of gellan gum depend on several factors such as the presence of cations, pH, temperature, and polymer concentration [103,106].

### 3.2. Protein-Based Films and Coatings

Proteins typically occur in two forms: fibrous proteins or globular proteins. Fibrous proteins are water-insoluble; otherwise, globular proteins are soluble in water and aqueous solutions of acids, bases, or salts. Protein coating-forming properties are better demonstrated in emulsified systems in which amphipathic proteins form films at oil/water or water/oil interfaces. The great barrier properties to polar gases and good adhesiveness to various materials make the proteins excellent materials to develop films and coatings. The distribution and amount of charged, polar, and non-polar amino acids along the protein chain create chemical potential. This potential can be adjusted for the specific requirements through chemical, enzymatic, and/or mechanical modifications of the different side groups attached to the central carbon in each amino acid [107].

The interactive forces between the polar and non-polar domains in the protein produce a cohesive matrix. Protein-based films and coatings are stabilized through electrostatic interactions, H-bonding, van der Waals forces, covalent bonding, and disulfide (S-S) bonds [108]. The occurrence of these interactions depends on the kind and arrangement of amino acids and protein structure. Fibrous proteins are associated with each other through H-bonding to made fibers, while in globular proteins, ionic, covalent, and H-bonds are present, which allow folding into complex structures [109]. The interactions in the protein-based films and coatings also depend on extrinsic factors such as processing temperature, drying conditions, pH, ionic strength, salt type, relative humidity during processing, and storage. Chain-to-chain interaction determines the strength of the film, higher interactions yield stronger films but are less permeable to vapors, liquids, and gases [110]. Protein-based films and coatings are often more stable than their polysaccharide and lipid counterparts and have a longer lifetime [108,111]. To address the replacement of films and coatings from petroleum sources, proteins from animal (casein, whey, collagen, gelatin, keratin, and egg albumen), and vegetal (soy, corn zein, and wheat) sources have been widely used (Table 2, Figure 1). Furthermore, it is believed that coating-based protein provides additional dietary value to the coated food [112], particularly due to the presence of essential amino acids [113].

#### 3.2.1. Whey Protein-Based Films Coatings 

Whey is a by-product of the cheese-making process, and whey proteins are technically defined as those that remain in the milk serum after coagulation of the caseins at pH 4.6 and 20 °C [123]. Whey protein is comprised of several individual proteins, such as β-lactoglobulin, α-lactalbumin, bovine serum albumin, and immunoglobulins [124]. Depending on the protein content, the powder is called whey protein concentrate (25–80%) or whey protein isolate, which contains >90% protein on a dry-weight basis [123].

Whey protein films are transparent, bland, and flexible, and have good mechanical hindrance and gas barrier properties at low relative humidity [125]. The interactions in films and coatings of whey protein are electrostatic forces. However, films made from thermal-denatured whey protein are stronger, more cohesive, and have better barrier properties than those made from native protein. Due to the β-lactoglobulin is thermolabile. After thermal denaturation, the interactions mentioned above occur in the new exposed groups; in particular, the thiol group in cysteine 121 is exposed and available for intermolecular S-S bonds formation [21,125]. However, due to the hydrophilic character of whey protein, these films have some limitations to moisture [63]. The incorporation of lipids (fat, oils, and waxes) [114,126] and nanoclays [127] is used to reduce the water vapor permeability of protein films. Furthermore, the addition of pectin and transglutaminase improves the mechanical and barrier properties of whey protein coatings [128]. An advantage of the use of whey protein is the obtention of bioactive peptides by trypsin hydrolysis with exhibited antibacterial activity against *Listeria monocytogenes* and *Staphylococcus aureus* (minimum inhibitory concentration 10–20 mg/mL) [129]. Henceforth, these peptides could act as bioactive compounds in whey protein-based coatings.

#### 3.2.2. Casein-Based Films and Coatings 

Caseins constitute ~80% of milk proteins in bovine milk. This is an abundant, low-cost, and commercially available food-grade additive [130]. α, β, and κ-casein together form colloidal micelles in milk containing large numbers of casein molecules stabilized by calcium-phosphate bonds [131,132]. Caseins form films from aqueous solutions due to electrostatic and hydrophobic interactions. The amphiphilic nature of the caseins makes them suitable for encapsulating hydrophobic compounds [133]. Usually, casein-based coatings with bioactive compounds consist of nanoparticles applied by casting or spraying methods [116]. 

The hydrophilic properties of these proteins limit the barrier and mechanical properties in the edible films and coatings. Therefore, different alternatives such as the incorporation of lipids [134], the combination with other hydrocolloids [135], and the increase the cross-linking through physical, chemical, or enzymatic treatments [136,137,138], have been assessed. Caseins are stables at 100 °C and 100 MPa [131]; however, as in whey protein, the heat promotes the formation of the intermolecular S-S bonds. This property has been used to improve the properties of casein films and coatings. Moreover, gamma-irradiation produces bityrosine bonds between protein chains, improving the mechanical, structural, and barrier properties of casein-based films and coatings [139]. 

#### 3.2.3. Collagen- and Gelatin-Based Films and Coatings

Collagen is a complex, fibrous, and water-insoluble protein. It is the major structural component of white connective tissue fibers, representing almost 30% of the total protein in invertebrate and vertebrate animals [113]. Gelatin is a high-molecular polypeptide obtained by partial hydrolysis of collagen extracted from bones, skin, and tendons of pigs, cows, and lambs. One hydrocolloid of low cost and high availability can be used as a gelling, thickening, or stabilizing agent in the food industry [113,140]. The basic mechanism of gelatin gelation is the random coil-helix reversion [141]. Films and coatings produced from collagen or gelatin exhibit good transparency, adherence to the food products, mechanical and barrier properties against oxygen and carbon dioxide, and can be manufactured by an extrusion or dipping process. Otherwise, these coatings are an excellent carrier of active compounds [142] or as encapsulating matrices to organic compounds or probiotic cells [143,144]. A disadvantage of gelatin is that can absorb 5 to 10 times its volume of water, contributing to the film’s instability [63,118,145]. Moreover, gelatin-based films and coatings start to melt from 27 to 34 °C, thus melting in the mouth. These facts are undesirable properties in foods that should be stored under high relative humidity before reaching consumption maturity [113]. 

#### 3.2.4. Egg Protein-Based Films and Coatings

Egg albumen is a globular protein that represents the second major component of liquid egg white, constituting up to 10.5% of the total weight of the liquid egg white. Albumen comprises five protein fractions ovalbumin, ovotransferrin, ovomucoid, ovomucin, and lysozyme. Ovalbumin is a 44.5 kDa protein that contains four free sulfhydryls (SH) groups available for cross-linking and is a random coil polypeptide conferring good film-forming properties of egg white. Ovotransferrin, ovomucoid, and lysozyme contain many S-S bonds [22,112]. Otherwise, lysozyme has been widely studied due to its antimicrobial activity, particularly against Gram-positive bacteria [145]. 

Egg albumen proteins are thermolabile and form strong heat-set gels. Hence, most methods for preparing egg-white films and coatings include the denaturation of proteins by adjusting the solution to pH 10.5 to 11.5 and heating at 40 °C for 30 min. The increase of SH groups with thermal and alkaline denaturation allows the formation of inter- and intra-molecular S-S bonds by oxidation and sulfhydryl-disulfide interchange reactions making more stretchable films [146]. During heat denaturation, ovalbumin, ovotransferrin, and lysozyme form stable intermolecular β-sheets structures. The denaturation of egg albumen proteins can be affected by the pH, salt concentration, sucrose, and temperature [22]. Changes in the conditions of these parameters have been used to improve the mechanical and barrier properties of egg albumen films and coatings. In line with this, the increment of the drying temperature causes protein denaturation, which induces tight networks and reduces film permeability. Otherwise, the addition of glycerol improves protein molecules’ movement and increases permeabil ity [147]. Moreover, the addition of lipids, such as unsaturated oleic acid, increases the tensile strength and elongation at break and reduces the water vapor permeability [148]. Albumen has good emulsifying and gelling properties, making it an ideal material for microencapsulation through the coacervation process [149,150]. 

#### 3.2.5. Wheat Gluten Protein-Based Films and Coatings

Gluten proteins are the storage proteins of wheat; they are an inexpensive, cohesive, viscoelastic proteinaceous material with filmogenic properties obtained as a by-product of the isolation of starch from wheat flour [151,152]. The functional properties of gluten are related to the gliadins and glutenins presents in wheat endosperm. Gliadins and glutenins are the water-insoluble fractions of the wheat proteins; gliadins are soluble in 70% ethanol. Gliadin is considered the solvent for glutenin; therefore, it is the main component responsible for the viscosity of wheat gluten. Otherwise, glutenin is the fibrous fraction in wheat gluten, providing elasticity and firmness [152,153]. For this reason, wheat gluten films have higher elasticity, an excellent barrier against oxygen, a high hydrophobic surface, and good thermal stability in comparison to other protein films [154]. However, the purity of gluten affects the appearance and mechanical characteristics of the films; wheat gluten with high purity forms more stable and clearer films [155]. The stability of these films is also affected by the method applied for its obtention; spray-dried films are more stable than films made by the dipping process [156].

The main drawbacks related to the use of wheat gluten-based films are their water sensitivity and low mechanical strength. These properties are highly affected in wet conditions due to water sorption and subsequent plasticization [154,157]. Lipid incorporation is expected to increase the hydrophobicity, decreasing the water sensitivity of these films without compromising the edibility [158]. Moreover, the addition of lignin nanoparticles (3%) into wheat gluten films increased the tensile strength (141.8%), young modulus (206.4%), and glass transition temperature (18.4%), and decreased the water uptake (37.7%) [159]. Otherwise, the addition of transglutaminase (20 units/g gluten) into wheat gluten films enhanced the tensile strength (~1 MPa), and the surface hydrophobicity increased from 88° to 113.08° [160]. 

#### 3.2.6. Soy Protein-Based Films and Coatings 

Soy protein is a mixture of globular proteins. β-conglycinin (7S globulin) and glycinin (11S globulin) are the two main globular proteins in soy protein, representing 37 and 31%, respectively. Conglycinin (140–170 kDa) consists of various combinations of three heavily glycosylated subunits. Glycinin (340–375 kDa) is made of six subunits linked via S-S bonds and is a great gelling, emulsifying, and foaming agent [161,162]. Heat and alkaline conditions can denature soy proteins, affecting film formation. Similar to β-lactoglobulin in whey protein, glycinin forms intermolecular S-S bonds when denatured, affecting the tensile properties of a film. β-conglycinin is less heat stable than glycinin; the proteins have denaturation temperatures between 70 and 80 °C, respectively [163]. Soy protein association and stability are pH- and ionic-strength-dependent [164].

Soy protein is available in three different forms according to the soy protein content as soy flour (54% protein), soy protein concentrate (SPC, 65–72% protein), and soy protein isolate (SPI, ≥90% protein) [165]. The high content of protein in SPI allows obtaining films with better properties than soy flour or SPC [166]. SPI films are clearer, smoother, and more flexible compared to other plant protein-based films, and have better gas barrier properties compared to films obtained from lipids or polysaccharides [112]. However, a large number of polar amino acids in SPI provide high hydrophilicity, resulting in a poor water vapor barrier and insufficient mechanical properties [167]. Different alternatives such as pH modification, fractionation by molecular weight, protein denaturation by temperature in alkali solutions, and blending with other compounds have been used to improve the drawbacks of SPI-based materials [168]. The addition of galactomannans to the film-forming solutions decreased the moisture content, total soluble matter, swelling in water, and hydrophilicity in comparison to coatings made with SPI [169]. 

#### 3.2.7. Corn Zein-Based Films and Coatings

Corn production reached 1147.62 million tons in the world during 2018 [170]. The protein content in corn ranges from 6 to 12% (dry basis) according to the variety. About 75% of the protein is contained in the endosperm tissue. Zein belongs to a class of proteins known as prolamines. This protein determines the hardness of corn endosperm; the most prevalent form is the α-zein. Zein has poor nutritional quality due to its deficiency in essential amino acids, such as lysine and tryptophan; moreover, the high content of non-polar amino acids provides a hydrophobic nature [171]. Hence, it is water-insoluble but is easily dissolved in organic solvents, such as ethanol (70–80%) or acetone. The solvent used, the temperature of solubilization, and the roughness of the coated surface strongly affect the morphology of the films. The use of cross-linking agents, such as succinic anhydride, eugenol, citric acid, and polyethylene glycol also affects the microstructure of the films and can be used to improve the mechanical properties and reduce the water vapor transmission of zein films and coatings [122,172,173].

### 3.3. Lipid-Based Films and Coatings

Lipids are apolar compounds that have low water-vapor permeability, making them great barriers against moisture migration and are useful for controlling food desiccation [63,172]. The natural hydrophobic compounds commonly used to coat fruits and vegetables are originated from animals, insects, and plant sources. These compounds are grouped into fats, oils, waxes, resins, and EOs (Figure 1). 

Some lipids have permeability values close to plastic films, such as low-density polyethylene or polyvinyl chloride. The permeability of solid lipids is usually lower than liquid lipids. Each hydrophobic substance has its physicochemical properties, and thus, edible films based on lipids have variable behavior against moisture transfer. The polarity of lipids depends on the distribution of electrostatic potentials on the molecules, chemical groups, aliphatic chain length, and the presence of unsaturation [112,174]. 

Usually, lipids are mixed with hydrocolloids by an emulsion technique [175] or by depositing lipid layers onto the surface of the pre-formed hydrocolloid film to obtain a bilayer coating [176]. The hydrocolloid incorporation produces an increment in the moisture permeability compared to the pure lipid [177] (Table 3). However, it has been reported that films and coatings that contain lipids cause damage to the appearance and gloss of the coated food products [178,179]. 

#### 3.3.1. Oil- and Fat-Based Films and Coatings

Fats and oils are obtained from animals and plants, mainly composed of triglycerides [15]. The physical and chemical characteristics of oils and fats greatly depend on the kind and proportion of the fatty acids in the triacylglycerol. An important feature common to most plant-origin oils and fats is the high percentage of unsaturated fatty acids in the triacylglycerols, making them more susceptible to oxidative deterioration [188,189]. It is important to take into consideration the composition of fatty acids of fats or oils, to identify their characteristics and determine the possible adulteration, as well as to know the physical and chemical properties of these compounds [190,191]. Vegetable oils act as carriers of fat-soluble vitamins (A, D, E, and K) and provide essential fatty acids such as linoleic and linolenic acid [192]. Moreover, the addition of hydrocolloids can improve the coating properties; for example, the incorporation of methylcellulose to palm oil makes an edible coating capable to maintain the quality of sapota fruit up to 7 days at 24 °C [193]. 

#### 3.3.2. Essential Oils-Based Films and Coatings

EOs are secondary metabolites rich in hydrophobic and volatile compounds. Moreover, these compounds have great antimicrobial activity attributed to terpenoids, terpenes, phenylpropanoids, and other aromatic compounds contained in them [194]. This activity can be improved by increasing the concentration of EO in the EC composition, but this concentration should be optimized to avoid alterations in the sensory properties of food products or the mechanical properties of the films [195]. Nevertheless, their strong organoleptic properties, low water-solubility, low stability, high susceptibility to environmental conditions, and high volatility limit their use. Henceforth, the application of Eos using an encapsulation system in a suitable delivery structure compatible with the food product offers a viable solution for such limitations. Emulsions, liposomes, and solid lipid nanoparticles are alternatives of encapsulation systems, being the most widely used emulsions on fresh and minimally processed foods [196,197]. 

#### 3.3.3. Wax- and Shellac-Based Films and Coatings

Waxes are compounds of high molecular weight and are the most efficient substances to reduce moisture permeability. Due to their high content in long-chain fatty alcohols and alkanes with long chains, their high hydrophobicity makes them insoluble in water and soluble in organic solvents [16,113,198]. Waxes reduce the surface abrasion during fruit handling and control soft scald formation (browning of the skin) in fruits such as apples by improving mechanical integrity and controlling the internal gas composition of the fruit [15]. The most common method for making wax microemulsions is the water-to-wax method. In this method, the water is added to the molten wax and/or resin in the presence of the fatty acid and a base to invert the emulsion to wax-in-water [198]. These formulations provide gloss to fruits and vegetables. However, their infrequent use in the food industry is related to their poor mechanical properties, oily appearance in some products, and tendency to lose gloss during storage of coated food products [199,200]. The common waxes used are carnauba, candelilla, and bee wax [112,201]. Fresh fruit and vegetables coated with waxes include apples, avocados, bell peppers, cucumbers, grapefruits, lemons, melons, passion fruit, peaches, pineapples, sweet potatoes, tomatoes, and yucca [175,201,202,203,204,205].

Shellac resin is a secretion from the insect *Laccifer lacca* Kerr, a versatile compound that dissolves in alcohol and alkaline solutions. Due to its compatibility with most waxes, shellac can be incorporated into wax formulations and contributes to the higher gloss of the coated food products [112]. Most Delicious apples marketed in the U.S.A. are coated with shellac or shellac in a mixture with carnauba wax [203]. Waxes and shellac added with Eos such as *Cinnamomum zeylanicum* have been used in commercial formulations to control citrus green and blue molds [206]. However, shellac has problems related to low gas permeability, which can lead to delayed ripening in some fruits and cause anaerobic conditions. Moreover, in the apple industry, shellac has further problems such as whitening or blushing, due to water condensation on the coated fruit surface after removal from cold storage. Nevertheless, shellac is recognized as a great coating to improve the appearance of apples [207].

## 4. Composite Films and Coatings

**Figure 2:**
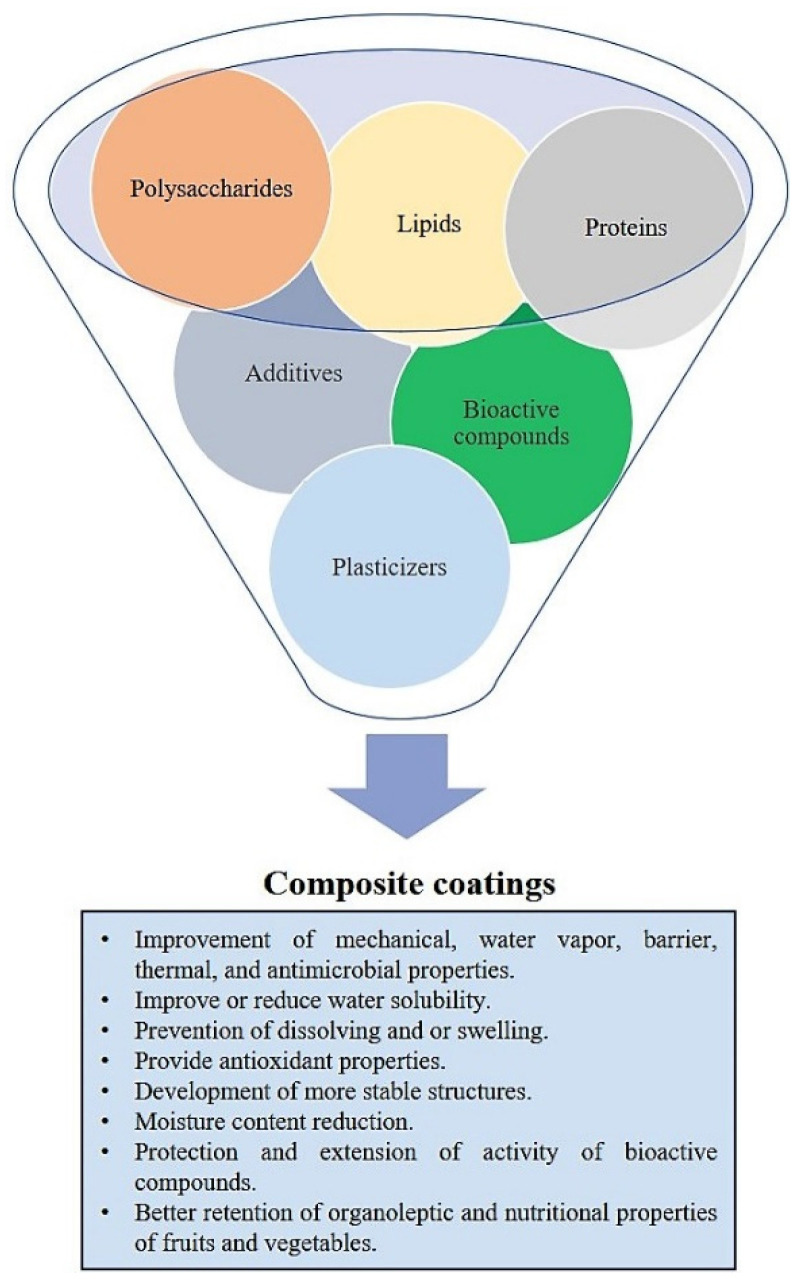
Composite coatings and their properties.

Nowadays, most research is carried out in the study of composite films and coatings to minimize the disadvantages of monocomponent films. In composite coatings, different substances are mixed to obtain new natural coatings with improved properties. The main objective of manufacturing composite films and coatings is to enhance moisture, gas barrier, and mechanical properties [63]. Composites films and coatings contain a combination of protein, polysaccharides, and/or lipids. Composites are divided into two categories: layer-by-layer composites or conglomerates. The layer-by-layer composites consist of two or more layers combined with the same or different coating material such as protein/protein (Table 2), polysaccharide/protein (Table 1), lipid/lipid, lipid/polysaccharides (Table 3), and others [208]. The structure of these multi-layered films optimizes the characteristics of the final film or coating, with a marked improvement of barrier properties (Figure 2). The main drawback of bi-layers films and coatings is the preparation method, which involves four stages: two dipping and two drying stages; this limits their application in the food industry. Furthermore, during the time storage, films are susceptible to develop cracks and exhibit non-uniform structure [208,209]. Otherwise, the conglomerates are created by mixing two or more biopolymers, yielding one homogeneous layer. The film developed has unique properties that combine the main attributes of each component [111,160]. 

## 5. Methods of Application of Natural Edible Coatings

The application of coatings is based on four technologies: deposition, adhesion, coalescence, and stabilization of the continuous coating layer through coacervation by drying, cooling, heating, or coagulation of the coating on the food surface [112]. Based on these technologies and depending on the fruit or vegetable, the most popular methods to coat fruits and vegetables are dipping, spraying, or brushing. These techniques exhibit several advantages and disadvantages, and performance mainly depends on the characteristics of the foods to be coated and the physical properties of the coating-forming solution (viscosity, density, and surface tension) [8,210]. Dipping is the most common method to coat fruits and vegetables, especially when the coating-forming solution is highly viscous [211]. Dipping is carried out by introducing the product for a time or several times to obtain multilayer coating between 5 and 60 s in the coating solution under controlled conditions [212]. The thickness of coatings is determined by the withdrawal speed. The dipping process allows to obtain uniform coatings around complex and rough surfaces and is highly adaptable to large-scale processes. However, the drying phase ideally would be performed in a cleanroom, to reduce the possibility of a build-up of residue or dirt onto the coating [210,213], and the coatings usually are thick [112]. Other drawbacks of the dipping method are that during the drying phase, the wet film is vulnerable to environmental factors (e.g., turbulent airflow), and the coating-forming solution in the tank is susceptible to contamination. Additionally, the material change from a liquid to a solid layer can lead to cracking in films, affecting the film properties [213]. 

Otherwise, when the coating solution is not highly viscous, the spraying method can be used. In this process, the food product is introduced into the coating system and is sprayed by controlling the final drop size of the spray solution. The properties of coatings obtained by spraying depend on the thickness of the spray gun, nozzle temperature, air, and liquid flow rates, the humidity of incoming air and polymer solution, drying time, and temperature [32,210]. A spray system increases the surface area of the liquid through the formation of droplets and distributes them over the food surface area through a set of nozzles. The main advantages of this technique are the obtention of uniform coatings and thickness control. Moreover, it is possible to obtain coatings combining hydrophobic and hydrophilic substances in two ways by applying an emulsion solution directly (formed before atomization) or by forming a bilayer after two spray pulverizations—for example, the application of sodium alginate, hydroxypropyl methylcellulose, ε-polylysine as the first layer, and calcium chloride solution as the second layer to form films and coatings [39,212,213]. Moreover, in spraying systems, the coating solution is not susceptible to contamination, allows temperature control of the solution, and can be easily automatized to continuous production [210]. Nowadays, there is an increased interest in the use of electrostatic spraying for the application of coatings in the food industry due to the advantages of material saving, high efficiency, and continuous industrial operation. The cost includes designing a specific atomizer according to the physical and rheological properties of each coating forming solution, which can limit the implementation of this system in the food industry [32].

Finally, the brushing method is used in some products, such as fresh beans and strawberries, when the reduction of moisture loss is an issue [8]. Brushing improves the spreadability of coatings over the fruit surface. The properties of coatings obtained by brushing vary according to the hair type and by the configuration of the tufts on the brush shaft [214]. Usually, it is complicated to apply on complex and rough surfaces, and the coatings obtained by brushing are thin, being prone to cracking, exposing the coated food [8]. Additionally, with this method, only one side of the fruit or vegetable is coated at a time, slowing the production process [35,112].

It is important to consider that the properties and efficiency of the films and coatings are affected by the application method; those obtained through evaporation have lower water vapor permeability than those prepared by spraying. Pectin- or alginate-based films prepared by evaporation of water-soluble components are subjected to redissolution in water or destruction in high humidity conditions [112]. Moreover, the efficiency of time storage also depends on the coating method used. The time storage of fruit or vegetables coated by brushing or spraying methods is lower compared to the dipping method [35]. Therefore, the selection of the appropriate coating method depends on the kind of food surface and the characteristics of the coating-forming solution. 

## 6. Trends and Challenges of Natural Films and Coatings

Edible films and coatings are used to increase the shelf-life of fruits and vegetables and retain their nutritional value and sensorial properties. In the last years, different bio-polymeric materials have been extracted from natural sources and used in manufacturing edible films and coatings for the preservation and improvement of the quality properties of fresh fruits and vegetables [6]. In agreement with this, films of 0.5 % (*w*/*v*) dextran produced by *Leuconostoc mesenteroides* SF3 and 1% (*w*/*v*) chitosan were developed for the packaging of mushrooms. The mechanical and barrier properties of chitosan-based films were improved by the addition of dextran and contribute to the maintenance of appearance and physicochemical features of mushrooms for up to 28 days at 4 °C [215]. Moreover, detranx of low molecular weight, called oligodextran, with prebiotic potential has been mixture at different concentrations with chitosan to develop composite films. The authors found that films with 0.5% (*w*/*v*) oligodextran and 1% (*w*/*v*) chitosan produced materials with a smooth topography and a homogeneous surface, which could have further applications in the development of food packaging [216]. Additionally, chitosan has been mixed with silk fibroin extracted from the waste of the MUGA cocoon to create a new composite coating. The developed material has improved thermal properties and hydrophobicity, extending the shelf-life of bananas by over 7 days at 25 °C, contributing to the maintenance of the initial weight, optical property, and firmness properties [217]. The use of microorganisms for the obtention of biopolymers is a great alternative to obtain compounds with unique properties varying the fermentation conditions. Otherwise, the use of agro-industrial wastes for the obtention of polymers in addition to providing new materials, contribute to reducing their negative impact on the environment.

However, these materials have several drawbacks, thus requiring the addition of additives to improve the physical and mechanical properties of the resulting packing [6]. In line with this, the incorporation of natural substances with antimicrobial properties, such as herbs, has been widely studied. The most common herbs used are *Aloe vera* gel, cinnamon, rosemary, tulsi, grapefruit, and thyme. Recently, *Aloe vera* gel, an aqueous liquid extracted from *Aloe vera* leaves, has generated great interest as an EC material, due to its antimicrobial and antifungal properties; moreover, it reduces the loss of moisture and water to extend the shelf-life of fruits [36,63,218]. Several studies have assessed the use of composite coatings based on biopolymers and *Aloe vera* gel to protect fruits such as grapes, apples, and mango fruit [28,30]. In mango fruit (cv. White Chaunsa), a coating based on chitosan/*Aloe vera* gel minimized the incidence of decay, reducing the weight loss, respiration rate, and ethylene production in comparison to the control after the storage [30]. The use of cinnamon, rosemary, tulsi, grapefruit, and thyme in films and coatings can be through their Eos or extracts, which have great antimicrobial properties due to the presence of phenolic compounds [206,219]. However, to maintain and improve their properties, it is necessary to use a polymeric matrix to emulsify or encapsulate the extracts, protecting their compounds from environmental factors such as pH, oxygen, light, and temperature [220]. For example, the use of gum guar improved the Neem extract properties and decreased the weight loss, and slowed down the changes in the chemical composition of Nagpur mandarin fruit during cold storage [219]. 

Peptides are short amino acid chains (2 to 100) positively charged with amphiphilic properties isolated from microorganisms, insects, plants, amphibians, birds, fish, and mammals to their use in the food industry [221]. A mixture of peptides produced by *Lactobacillus plantarum* and *Lactobacillus lactis* subsp. *Lactis* were used to coat pineapple slices, decreasing the amounts of *Escherichia coli*, *Salmonella*, and *Shigella* after 5 days of refrigeration [218]. Therefore, the incorporation of peptides with antifungal and antimicrobial properties into films and coatings brings a new possibility to the use of other natural sources to extend the postharvest shelf-life of fruits and vegetables. Some studies have demonstrated that the incorporation of biocontrol agents such as yeasts or bacteria into the coating matrix contributed to preventing fungal decay in tropical fruits, extending their shelf-life [10,15], and improved the thermal and barrier properties of sodium alginate films [222]. The polysaccharides acquired from microbial resources, such as pullulan, curdlan, scleroglucan, dextran, and xanthan, have obtained more attention due to their efficacy to coat foods. Regarding commercialization, microbial polysaccharides are highly available and have low manufacturing prices. Moreover, the obtention of microbial polymers can be easily scaled up via manipulation of the environmental conditions or through genetic modification [63]. The polysaccharides from microbial sources improve the postharvest practices, resulting in loss reduction and shelf-life extension of fruits and vegetables, providing higher benefits for growers and consumers [35]. Herbal extracts and peptides enhance the nutritional properties of the coated food products by adding antioxidants, functional ingredients, and amino acids, which can include essential amino acids.

Another important approach in the development of biodegradable films and coatings is bioplastic produced by microalgae such as *Chlorella* and *Spirulina*. Algal biomass is a sustainable source of biopolymer extraction since it does not compete with the food source. Algal biopolymers (starch, poly-hydroxybutyrates, and so on) can be obtained by the extraction of biopolymers or by the composite preparation employing algal biomass, plasticizers, and additives, followed by mechanical/physical extrusion [223]. In line with this, *Chlorella* sp. Has been used in a mixture with pomegranate seed oil to extend the self-life of umbu (*Spondias tuberosa*) fruit. The formulated material contributes to the maintenance of the content of vitamin C and phenolic compounds, reducing the loss of weight and firmness and delaying the ripening process in fruits stored at 14 °C for 12 days [224]. However, the characterization of the developed coatings was not carried out.

The modification of film properties can also be achieved by linking the biopolymer to other compounds. For example, the features of chitosan coatings were improved by covalent linkage of bioactive volatiles such as vanillin and *trans*-cinnamaldehyde by Schiff base reaction. This reaction produced a new polysaccharide with self-assembling ability, overcoming the problem of solubility related to the incorporation of active lipophilic agents in aqueous media, and prevented the volatilization of the compounds, thus neutralizing their distinctive odors, facilitating their use. Moreover, the coating forming solution developed did not produce an adverse effect on the properties of fresh-cut melon and reduced the total microbial, mold, and yeast counts after 14 days of storage at 7 °C [225].

On the other hand, the use of nanotechnology is leading to the development of a new generation of edible coatings. The nano-systems allow to control the release of active compounds under specific conditions, display an increased surface region, and the submicronic structures have higher distribution and homogeneity on the coated surface [63,226]. For example, the use of nanoemulsions provides physical stability to Eos, increases their bioactivity, and minimizes the influence of their organoleptic characteristics on food products [227]. Moreover, they allow dispersing Eos in water to obtain homogenous coating forming solutions and improve the wettability on the food surface [18,228]. On the other hand, electrospinning is used to form coatings on the surface of fruits using a high-voltage electrostatic force. In this method, soft nanofibrous membranes are applied to food to offer gentle protection. Coatings obtained by electrospinning can retain freshness, solving the storage and transportation problems of some fruits, such as cherry tomatoes and kumquat [29,229]. In line with this, nanofiber of carboxymethyl chitosan/polyoxyethylene oxide prevents weight loss and postharvest diseases in strawberries, remaining non-toxic and harmless [29]. Otherwise, nanofibers of corn zein/titanium oxide delay the ripening process in cherry tomatoes by ethylene photocatalysis [122]. Coatings obtained by electrospinning can be formed by single-strand fiber spun with a single-axis needle, while multiaxis needles can be used to spin multicomponent fibers at the same time. The properties of the nanofibers obtained depend mainly on three factors: the electrospinning fluids, the operating conditions, and the environmental factors. The control of the whole parameters and the high cost of the equipment represents the main disadvantages of this technique [229,230].

Linked to the increasing application of nanotechnology to coat food is the development of smart films and coatings. This means that the coating system provides to the consumer information about the conditions of the food or its environment (e.g., temperature and pH) [231]. To achieve this, the use of nanoparticles with a core of anthocyanidin or sodium acetate into a chitosan matrix has been proposed. The addition of the microparticles enhanced mechanical and barrier characteristics; improved the antibacterial, antifungal, and antioxidant properties; and provided UV-protective activity in comparison to chitosan films without microparticles. Moreover, they served as time-temperature indicators in coated cheese. To make this possible, the particles developed must have a glass transition temperature beyond the temperature range acceptable for the storage of the assessed food. When the storage temperature exceeds the value, the nanoparticle changes from glass to viscous flow state, favoring the mixture of anthocyanin and sodium acetate, resulting in the formation of the anionic purple-colored form of anthocyanin [232]. However, the use of this technology is still to be assessed in fruits and vegetables; further investigation should be carried out.

The use of natural films and coatings is still limited by the high availability and low cost of synthetic food packaging. Moreover, inconsistent results are often reported, depending on the source and concentrations of the biopolymers used. In addition to this, the behavior of each coating varies depending on the crop, storage conditions, and phytopathogen fungi assessed. In other cases, the full characterization of a film is given, but its impact on the properties of fresh food is not reported. Otherwise, the films and coatings are sometimes added with bioactive agents without their full chemical composition and identification of the main compounds [219]. Moreover, another important fact that usually has not been assessed is the effect of the interaction of food components with the films, even when it has been demonstrated that starch, whey proteins, and sodium chloride affect the antimicrobial properties of chitosan [233]. These facts have a great impact on the decision of producers to choose a natural and recently developed formulation to coat their food products. Therefore, scientists and the food packaging industry should work to provide more consistent information about the features of the films and their impact on the properties of coated foods. Moreover, further investigation should be focused on the obtention of polymers from agro-industrial wastes and in the development of composite coatings aimed to satisfy the main challenges in this area, the maintenance of food quality properties, and delay of the microbial decay.

## 7. Conclusions

Edible food packaging based on materials obtained from natural sources represents a low-cost alternative to reduce the losses of fruits and vegetables and the pollution by synthetic packaging. Moreover, the obtention of these polymers can contribute to reducing agro-industrial waste. However, the use of biopolymers has some disadvantages that should be enhanced by their mixture with other hydrocolloids or by the addition of cross-linking, antioxidant, antimicrobial, and antibrowning agents. Due to each food having a different composition and storage conditions, any formulation aimed to develop films and coatings must be optimized considering the mechanical, barrier, thermal, and antimicrobial properties to satisfy the requirements of the coated foods, to achieve the shelf-life extension maintaining their nutritional and organoleptic properties. However, even considering the research carried out in this area, there are still many biopolymers and additives with good characteristics to form active and smart edible films and coatings that have not been explored in detail. These biopolymers would contribute to the successful replacement of synthetic coatings for the protection and preservation of fresh food products. Therefore, the development of natural composite coatings represents the more focusing area of scientists in this research field and the packaging industry. Finally, it is important to consider the method used to develop the food packaging, because mechanical and gas properties are strongly affected by the application method. In this field, more research is required to satisfy the global demand for minimally processed foods.

## Figures and Tables

**Table 1:** Polysaccharide-based coatings for different fresh foods.

Polymer Matrix	Additives	Biocontrol Agent/Bioactive Compound	Method of Application	Fruit/Vegetable	Significant Function	Reference
Carboxymethyl cellulose	Glycerol, Tween 80	*Mentha spicata* EO	Dipping	Strawberries	Extension of the shelf-life for at least 12 days, delaying the weight loss, titratable acidity, and pH, microbial spoilage (yeast, molds, psychrotrophic bacteria, total viable count), and *Listeria monocytogenes*. Moreover, it had positive effects on water vapor resistance and the respiration rate of coated strawberries.	[23]
Carboxymethyl cellulose	Glycerol	-	Dipping	Strawberries	Reduction of the weight loss and decay, and preservation of the concentration of ascorbic acid and total phenolic content of strawberries during 16 days of storage at 4 °C.	[24]
Cellulose nanofibers	Sucrose ester fatty acid	Oleic acid	Brushing	Bananas	Delay of the ethylene biosynthesis pathway and reduction of the production of ethylene and CO_2_, preservation of the fruit surface morphology to provide more uniform coating coverage. Effectiveness for reducing chlorophyll degradation of banana peels and weight loss, maintenance of firmness of fruit, thus enhancing the marketability and storability during ambient storage (20 °C for 10 days).	[25]
Hydroxypropyl methylcellulose	- ^a^	*Origanum heracleoticum* L. EO	Dipping	‘Formosa’ plum	Reduction in the respiration rate, ethylene production, total weight loss, firmness maintenance, less surface color change, and total cell count, compared to uncoated plums after 14 days of storage at 23 °C.	[26]
High methylester pectin	Sunflower lecithin, sucrose monopalmitate	Biosecur F440D, EOs mixture	Dipping	Pre-cut carrots	Improvement of the shelf-life, stabilization of polyphenolic and terpenic compounds during time storage. Antimicrobial activity against *Listeria monocytogenes* and *Penicillium chrysogenum*.	[27]
Banana starch, chitosan	Sorbitol	*Aloe vera* gel	Dipping	Strawberries	Reduction of fungal decay and increase of the shelf-life up to 15 days of storage, decreasing the water vapor loss from fruit and the structural decay.	[28]
Carboxymethyl chitosan, polyoxyethylene oxide	-	-	Electrospinning	Strawberries	Reduction of the weight loss, prevention of diseases and rot, and improvement of the appearance of fruit in the storage at room conditions for 9 days, while remaining non-toxic and harmless.	[29]
Chitosan	Tween 80, glycerol	*Aloe vera* gel	Dipping	White Chaunsa mangoes	Delay of the postharvest decay incidence and retention of quality attributes such as titratable acidity, total soluble solids, firmness, weight loss, and peel color of the fruit during storage. Inhibition of the respiration rate and ethylene production, and increase of the ascorbic acid, total phenolic content, and antioxidant activity of mango fruit during storage.	[30]
Chitosan, canola oil	Glycerol	*Byrsonima crassifolia* extract (L.) Kunth	Spraying	Bell pepper	Increase in the content of carotenoids and antioxidant capacity decrease the weight loss and the change of color after 21 days of storage; the microbiological activity was reduced by 85%.	[31]
Chitosan	Acetic acid, glycerol	-	Electrostatic spraying	Strawberries	Reduce the physiological and biochemical changes of fresh strawberries to prolong the shelf-life during cool storage (15 days at 4 °C) compared to uncoated treatment.	[32]
Sodium alginate	Glycerol	*Meyerozyma caribbica*	Dipping	‘Hass’ avocado	Control of anthracnose produced by *Colletotrichum gloeosporioides* during the postharvest stage and decrease of the weight loss during the time storage (20 days; 10 days at 6 °C and ripening at 25 °C).	[33]
Sodium alginate	Calcium chloride	Cyclolipopeptides produced by *Bacillus subtilis*	Dipping	Blueberries	Antifungal activity and freshness-maintenance properties, the incorporation of cyclolipopeptides in a sodium alginate film provide an easy-cleaning, multifunctional coating film for vulnerable berries.	[34]
Gum guar	-	Neem extract	Dipping	Nagpur mandarin	Spoilage inhibition, decrease in weight loss, retention of acidity, total soluble solids, ascorbic acid content, and better organoleptic properties after cold storage.	[34]
Pullulan	-	-	Dipping	Bananas	Significant weight reduction, vitamin C, browning index, peel to pulp ratio, and soluble solids content. Moreover, increase of the firmness and high total and residual sugar contents, contrasted with uncoated (control) fruits, the extension of the shelf-life of banana up to 20 days of storage at 25 °C.	[35]
Xanthan gum	Calcium chloride, glycerol	-	Dipping	Pinot noir grapes	Decrease in the weight loss, suppression of polyphenol oxidase, ascorbic acid oxidase, polymethyl esterase activities, higher phytochemical contents, and maintenance of the structural integrity of the grape during the 21 days of storage at 4 °C.	[36]
Gellan gum	Glycerol, sunflower oil	-	Dipping	Apricots	Preservation of the carotenoids content, biochemical characteristics, external color, weight loss, and increase in the firmness compared to the uncoated fruits. Moreover, reduction in the peroxidase and polyphenol oxidase activities after 15 days of storage at 4 °C.	[37]

^a^ No added; EO: essential oil.

**Table 2:** Protein-based coatings for different fresh fruit products with its functions.

Polymer Matrix	Additives	Biocontrol Agent/Bioactive Compound	Method of Application	Fruit/Vegetable	Significant Function	Reference
Whey protein concentrate	Glycerol	*Salvia officinalis* L. extract	Dipping	Pistachio kernels	Inhibition of *Aspergillus flavus* growth and aflatoxins production in pistachio kernels after 9 days of storage at 20 °C.	[114]
Whey protein isolate	Glycerol, trehalose	- ^a^	Nanofibers applied by dipping	Fresh-cut apples	Retention of phenolic content, weight loss reduction, and browning inhibition in fresh-cut apple. Coatings extended the shelf-life of apple pieces and retarded the senescence process compared with that of apple pieces used as controls after 10 days at 4 °C. Moreover, the coatings did not influence consumer acceptance.	[115]
Bovine casein	-	Eugenol	Nanoparticles applied by spraying	Pear fruits	Complete suppression of anthracnose disease in pears after 8 days of storage at 25 °C.	[116]
Gelatin from porcine skin, zein corn	Glycerol	Propolis extract	Nanoparticles applied by dipping	Raspberries	The encapsulation controlled the release of the extract and extended its efficiency over time. Inhibitory effect on *Penicillium digitatum* and *Botrytis cinerea* in the fruit after the storage (11 days at 5 °C).	[117]
Wheat gluten	Glycerol, ethanol	-	Dipping	Fresh-cut pineapples	The coated slices had a firmer texture, less juice leakage, and lower counts of psychrotrophic, mesophiles, molds, and yeasts than uncoated slices. *Salmonella*, *Escherichia coli*, and total coliform were not detected after 12 days of storage at 5 °C.	[118]
Soy protein isolate	Glycerol	Limonene	Dipping	Persian lime	Low incidence of *Penicillium italicum* disease in the fruits. Reduce the water losses and maintain the color in coated limes in comparison to uncoated limes, and controlled the liberation of the active agent during the storage (13 days at 13 °C).	[119]
Soy protein isolated	-	Lemon extract	Dipping	Fresh-cut melon	Reduction of the total plate count and yeast and molds amounts. Effective to preserve vitamin C and color, retardation of the respiration. Water loss reduction, thereby protecting the cut tissue of melon samples from wilting, and sensory attributes were preserved to a remarkable extent after 12 days of storage at 4 °C.	[120]
Corn zein	Polyethylene glycol 400	-	Dipping	Fresh-cut apple	The films shown antioxidant characteristics, which delay the browning, maintenance the freshness, and prevent weight loss in apple slices stored at 20 °C for 24 h.	[121]
Corn zein	Ethanol, titanium oxide	-	Electrospinning	Cherry tomatoes	Photocatalytic activity against ethylene reducing its concentration, delaying the ripening process after 22 days of storage.	[122]

^a^ No added.

**Table 3:** Lipid-based coatings for different fresh fruit products with their functions.

Lipid	Polymer/Additives	Method of Application	Fruit/Vegetable	Significant Function	Reference
Oleic acid	Cellulose nanocrystal, chitosan, acetic acid	Dipping	Green Bartlett pears	Reduction in the ethylene production and superficial scald of ‘Bartlett’ pears during the long-term cold storage (−1.1 °C for 6 months).	[180]
*Mentha* EO	Chitosan, acetic acid, Tween 80, glycerol	Dipping	Papaya fruit	Reduction of the development of anthracnose lesions caused by *C. gloeosporioides* and *Colletotrichum brevisporum* after 10 days of storage at 25 °C.	[181]
Tea tree oil	β-cyclodextrin, ethanol	Dipping	Cherry tomatoes	Control of the artificially induced *B. cinerea* infections and shelf-life extension of the fruits for 7 days at 20 °C.	[182]
Bee wax	Xanthan gum, polyvinyl alcohol, propilenglycol	Nanoparticles applied by dipping	Strawberries	Decrease of decay rates reflected in less fungal growth, weight loss, and physiological damage after 21 days of storage at 4 °C.	[183]
Bee wax	Hydroxypropyl methylcellulose, stearic acid, glycerol	Dipping	Guavas	Reduction of mass loss, maintenance of green color, and increase in firmness compared to the uncoated fruits after 8 days at 21 °C.	[184]
Carnauba wax, coconut oil, oregano EO	Tween 20	Brushing	Cucumber	Decrease the weight loss and reduction of microbial loads (mesophilic bacteria, molds, and yeasts) after 15 days of storage at 10 °C in comparison to uncoated fruit.	[185]
Carnauba wax, oleic acid, myristic acid	Glycerol monolaurate, ammonia	Dipping	Indian jujube fruit	Reduction of weight loss, respiration rate, and ethylene production and decreases the activity of polygalacturonase, pectin methylesterase, and cellulase delaying the flesh softening. Delay the skin color change and preservation of chlorophyll content and ascorbic acid providing better sensory quality compared to uncoated fruits after 12 days of storage at 20 °C.	[186]
Shellac	Ethanol	Dipping	Valencia oranges	Reduction in weight and firmness loss, and flavor quality preservation. The coatings dried quickly, forming a coating that was odorless and not sticky, and making the fruit glossy. The coatings did not show visible cracks after 60 days of storage at 5 °C.	[187]

EO: essential oil.

## Data Availability

The data presented in this study are available on request from the corresponding author.
